# Exploratory Analysis of Serum Oxidized Albumin as a Potential Prognostic Indicator for Diabetic Microvascular Complications: A Retrospective Cohort Pilot Study

**DOI:** 10.7759/cureus.83976

**Published:** 2025-05-12

**Authors:** Tomoya Kawaguchi, Yuka Kobayashi, Keiko Yasukawa, Kengo Miyoshi, Nagisa Ishibashi, Satoshi Usami, Yosuke Inaba, Masaya Sato, Makoto Kurano, Ryo Suzuki, Tomohisa Aoyama, Yutaka Yatomi, Toshimasa Yamauchi

**Affiliations:** 1 Department of Diabetes and Metabolic Diseases, Graduate School of Medicine, The University of Tokyo, Tokyo, JPN; 2 Department of Clinical Laboratory, The University of Tokyo Hospital, Tokyo, JPN; 3 Graduate School of Education, The University of Tokyo, Tokyo, JPN; 4 School of Medicine, Chiba University, Chiba, JPN; 5 Clinical Research Promotion Center, The University of Tokyo Hospital, Tokyo, JPN; 6 Department of Gastroenterology, The University of Tokyo Hospital, Tokyo, JPN; 7 Department of Diabetes, Metabolism and Endocrinology, Tokyo Medical University, Tokyo, JPN; 8 Graduate School, International University of Health and Welfare, Tokyo, JPN

**Keywords:** biomarker, diabetic complications, human mercaptalbumin, oxidative stress, pilot study

## Abstract

Introduction

Oxidative stress is known to play a key role in the pathogenesis of diabetic complications. However, a useful biomarker of oxidative stress has not yet been widely adopted in clinical practice. We explored whether the ratio of serum oxidized albumin (human non-mercaptalbumin, HNA) to total albumin (HNA%) is associated with the five-year prognosis of diabetic complications.

Methods

In this single-center, retrospective cohort pilot study, we evaluated participants with diabetes who had been hospitalized at baseline and regularly followed up as outpatients. We measured the HNA% at baseline and at a five-year follow-up and assessed the development or progression of diabetic complications. We examined the relationship between baseline HNA% and changes in diabetic complications.

Results

In participants with simple diabetic retinopathy at baseline, lower baseline HNA% was associated with shorter duration of diabetes and with five-year improvement of retinopathy, irrespective of follow-up glycated hemoglobin levels (25.46% vs. 33.71%, p = 0.021). Additionally, in the overall cohort and in the subgroup with baseline chronic kidney disease (CKD) stage G2, baseline HNA% above the cutoff value was associated with increased risk of CKD progression (overall: RR 1.61, p = 0.042, stage G2 only: RR 1.83, p = 0.047). Regarding macrovascular complications, follow-up HNA%, but not baseline HNA%, was related to their development.

Conclusions

These exploratory findings suggest that HNA% may have potential as an indicator of the five-year prognosis of diabetic retinopathy and diabetic kidney disease. Further prospective studies are warranted to validate these findings.

## Introduction

Diabetic complications pose a substantial and growing threat to both the quality of life and life expectancy of individuals with diabetes worldwide [1-5]. In the United States, diabetes was identified as the fourth leading cause of disability-adjusted life years in 2021, underscoring its escalating impact on morbidity and mortality [6,7]. Similarly, in Japan, diabetic retinopathy accounted for 12.8% of newly registered cases of visual impairment in 2015, ranking third following glaucoma and retinitis pigmentosa [8]. Furthermore, diabetic nephropathy remains the leading cause of both incident dialysis initiation and chronic dialysis dependence in Japan, highlighting the profound burden of renal complications associated with diabetes.

Oxidative stress is recognized as a key contributor to the pathogenesis of diabetic complications, with oxidized albumin emerging as a potential biomarker. Several molecular pathways have been proposed to explain how hyperglycemia induces cellular metabolic abnormalities, including the polyol pathway, hexosamine pathway, protein kinase C pathway, advanced glycation end-products pathway, and oxidative stress itself [9-11]. Although various oxidative stress markers have been investigated, there are no established biomarkers for oxidative stress in routine clinical use [12,13]. Albumin can be classified into reduced albumin (human mercaptalbumin, HMA) and oxidized albumin (human non-mercaptalbumin, HNA), and the ratio of oxidized albumin to total albumin (HNA%), calculated as HNA/ (HNA + HMA) × 100%, has been reported as a potential indicator of oxidative stress [14]. In 2017, a novel high-performance liquid chromatography method was developed, allowing for precise measurement of HNA% in approximately 12 minutes per sample [15]. In 2020, we reported cross-sectional associations between HNA% and the severity of diabetic retinopathy, neuropathy, and nephropathy in a cohort of 164 individuals with diabetes [16]. Furthermore, in 2021, a retrospective case-control study suggested that Cys-albumin may serve as a biomarker for the progression of kidney disease in individuals with type 2 diabetes [17].

However, it remains unclear whether HNA% can predict the development and progression of diabetic complications, and longitudinal associations beyond renal function have yet to be established. Therefore, we hypothesized that baseline HNA% may serve as a prognostic indicator for diabetic complications. This pilot study aimed to explore potential associations between baseline HNA% and subsequent changes in diabetic complications over a five-year period among individuals with diabetes who had participated in our previous cross-sectional study, with the ultimate goal of facilitating more effective and targeted future research.

This article was previously presented as a meeting abstract at the 59th Annual Meeting of the Japanese Society of Molecular Medicine on April 12, 2024.

## Materials and methods

This is a single-center, retrospective cohort pilot study conducted at the University of Tokyo Hospital, approved by the Ethics Committee of the University of Tokyo (ethics review number 2020357NI-(4)), in accordance with the Declaration of Helsinki. Written informed consent was obtained from all participants.

Study participants

The participants in this study were selected from a cohort of 164 individuals who had participated in a previous cross-sectional study conducted between 2016 and 2017 at the University of Tokyo Hospital [16]. All individuals in this cohort had been hospitalized in the Department of Diabetes and Metabolic Diseases for the treatment of diabetes, which served as the primary inclusion criterion. Patients who were pregnant, lactating, or experiencing acute organ failure (such as pneumonia, acute myocardial infarction, acute cerebral infarction, diabetic ketoacidosis, or hyperosmolar hyperglycemic state), as well as those with congenital cognitive impairment, were excluded from the original cohort.

For the present follow-up study, eligible participants were those who, as of April 2021, continued to receive outpatient care at the same institution. Individuals who were unable to communicate effectively in daily life or from whom blood samples could not be obtained for research purposes were excluded. Seventy-seven people (100%) provided written informed consent and were included in the final analysis.

Of the 77 study participants (100%), a variety of comorbidities and prior medical histories were reported. The most frequently observed conditions included fatty liver disease (n = 27; 35%), hyperuricemia (n = 18; 23%), appendicitis (n = 15; 19%), cataracts (n = 11; 14%), anemia (n = 11; 14%), and sleep apnea syndrome (n = 11; 14%). Other relatively common conditions were cholelithiasis (n = 7; 9%), atrial fibrillation (n = 5; 6%), herpes zoster (n = 5; 6%), and osteoarthritis (n = 4; 5%).

Several autoimmune, hepatic, gastrointestinal, and psychiatric conditions were also documented, including rheumatoid arthritis (n = 2; 3%), systemic lupus erythematosus (n = 1; 1%), gastroesophageal reflux disease (n = 3; 4%), chronic pancreatitis (n = 4; 5%), and depressive disorders (n = 2; 3%). A range of infectious and neoplastic conditions were also reported, such as tuberculosis (n = 4; 5%), gastric cancer (n = 3; 4%), colorectal cancer (n = 3; 4%), prostate cancer (n = 3; 4%), breast cancer (n = 3; 4%), and endometrial cancer (n = 2; 3%).

Less frequent but notable conditions included rare syndromes (e.g., Kartagener syndrome, Castleman disease, and Ebstein anomaly), organ transplants (n = 2; 3%), and congenital anomalies (e.g., Hirschsprung disease and ventricular septal defect). Overall, comorbidities and medical histories represented a broad clinical spectrum, including cardiovascular, musculoskeletal, endocrine, renal, hepatic, and dermatologic domains.

Assessment parameters

The following data were collected from medical records and interviews: age, sex, duration of diabetes, height, weight, BMI, smoking status (never/former/current smoker), family history of diabetes, Mini Mental State Examination (MMSE) score, diabetic neuropathy (based on subjective symptoms attributable to diabetic neuropathy, bilateral diminishment or absence of Achilles tendon reflexes, decreased bilateral medial malleolus vibration sensation, and evaluation based on coefficient of variation of R-R intervals in 12-lead electrocardiogram), diabetic retinopathy stage (no retinopathy/simple retinopathy/pre-proliferative retinopathy/proliferative retinopathy: based on modified Davis classification, evaluated by ophthalmologists within the past year), diabetic nephropathy (stages 1-5, based on the 2023 Classification of Diabetic Nephropathy by the Joint Committee on Diabetic Nephropathy and Classification Working Group), history of coronary heart disease, history of cerebrovascular disease, systolic and diastolic blood pressure, diabetes treatment status (no medication/oral hypoglycemic agents only/glucagon-like peptide-1 receptor agonist use/insulin use), antihypertensive medications, and lipid-lowering agents.

Deterioration of diabetic retinopathy was defined as a progression by one or more stages according to the modified Davis classification. Similarly, improvement of diabetic retinopathy was defined as a regression by one or more stages based on the same classification. Simple diabetic retinopathy (SDR) in this system corresponds to nonproliferative retinopathy as defined in the Early Treatment Diabetic Retinopathy Study classification.

Plasma specimens were utilized for glucose and glycated hemoglobin (HbA1c) measurements, and serum specimens were employed for the determination of albumin, total cholesterol, triglycerides, high-density lipoprotein cholesterol, low-density lipoprotein cholesterol, uric acid, blood urea nitrogen, estimated glomerular filtration rate (eGFR), creatinine, and HNA%. Detailed measurement methods of HNA% have been described in the previous report [15] and are omitted here.

HbA1c and eGFR values were collected from regular visits between baseline and the follow-up period, and a time-weighted average of HbA1c was calculated.

Statistical analysis

Statistical analyses were performed using JMP Pro 17^®^ for Windows (SAS Institute Inc., Cary, NC, United States). Receiver operating characteristic (ROC) curves were constructed using the R statistical computing environment (version 4.3.2). Descriptive statistics for continuous variables are presented as median (IQR), and discrete variables are shown as number (%). When comparing means between groups, values are presented as mean (SD). Unpaired two-sample comparisons were conducted using Student’s t-test, while paired comparisons used paired t-tests. Nominal scale data and unpaired ordinal scale data between two groups were compared using Pearson’s chi-square test. Paired ordinal scale data between two groups were analyzed using the Wilcoxon signed-rank test. Kaplan-Meier curves and log-rank tests were employed to assess time-to-event outcomes.

ROC curves were constructed to evaluate the predictive ability of baseline HNA% for chronic kidney disease (CKD) stage progression. The optimal cutoff value in the ROC curve was determined using the Youden index, which maximizes sensitivity (1-specificity).

CKD staging was determined using eGFR measurements from regular outpatient visits. To minimize fluctuations due to acute conditions during hospitalization, baseline CKD staging was determined using the average of three eGFR measurements after discharge. For survival analysis, stage progression in CKD severity classification was defined as the event [17]. In this study, an event was defined as the observation of one-stage progression from the baseline stage in two consecutive regular outpatient visits. This definition was chosen because we aimed to evaluate renal function deterioration due to diabetic kidney disease, not acute comorbidities or dehydration. For example, for a participant with CKD stage G2 at baseline, the event occurrence date was determined as the date of the second outpatient visit of the two consecutive visits, when stage G3a was first observed. As only participants who maintained their clinical follow-up were included in the study, there were no dropouts, enabling both survival and comprehensive analyses to be performed. We performed a subgroup analysis of CKD stage G2 participants to control baseline renal function differences.

## Results

Enrollment

**Figure 1:**
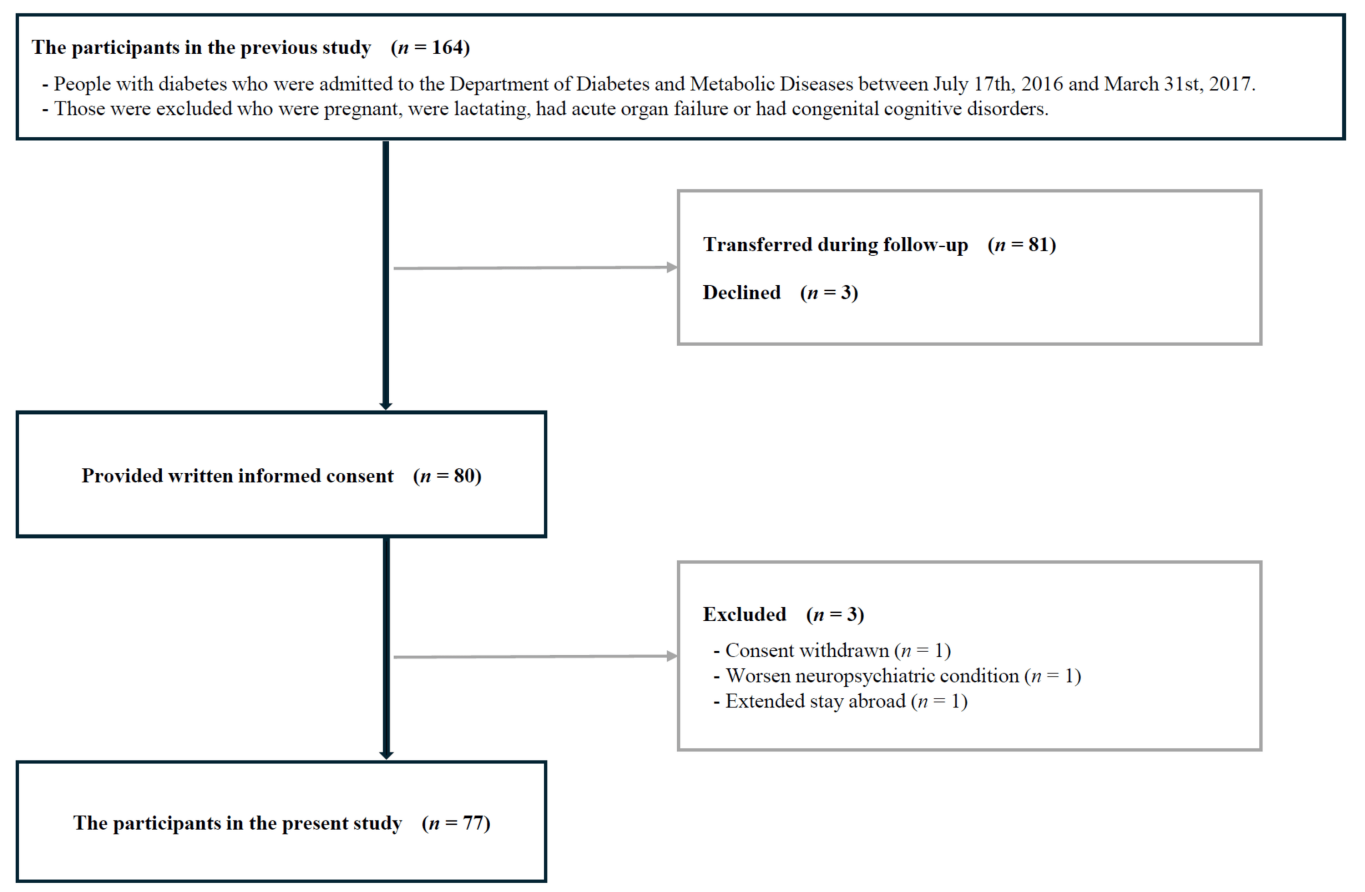
Flow diagram of the study participants A total of 77 individuals with diabetes participated in this study.

Of the 164 individuals who participated in our previous cross-sectional study, 81 had transferred to other hospitals by the start of the present study. Among the 83 people who continued treatment at our department, three declined participation. Of the 80 participants who provided informed consent, three were subsequently excluded for the following reasons: one withdrew due to refusal of ophthalmological examination, one had unstable neuropsychiatric conditions, and one was forced into a prolonged overseas stay due to the COVID-19 pandemic. The final study population comprised 77 participants (Figure 1).

Clinical characteristics of study participants

As shown in Table 1, 54 participants (70%) presented with neuropathy. Among the 71 people (92%) evaluated by ophthalmologists, 30 (39%) exhibited retinopathy of SDR or greater severity. Regarding nephropathy, 36 participants (47%) had urinary albumin levels below the threshold, 24 (31%) showed microalbuminuria, 15 (19%) presented with overt proteinuria, and two (3%) had reached chronic renal failure. The median HbA1c was elevated at 8.6%, reflecting the baseline inpatient status of the participants. The median age was 66 years, with 42 participants (55%) aged 65 or older, as shown in Table 1.

**Table 1:** Baseline characteristics of 77 study participants Values are expressed as medians (IQRs), and frequencies are presented as n (%). N = 77 (100%) unless otherwise specified. ^†^ Participants using medications from multiple categories were classified into the lowest category within the classification hierarchy. Nephropathy stage was determined based on the Classification of Diabetic Nephropathy 2014 proposed by the Joint Committee on Diabetic Nephropathy in Japan. Alb, serum albumin; BUN, blood urea nitrogen; Cre, creatinine; eGFR, estimated glomerular filtration rate; GLP-1, glucagon-like peptide 1 receptor agonists; HbA1c, glycated hemoglobin; HNA%, percentage of human non-mercaptalbumin; LDL-C, low-density lipoprotein cholesterol; MMSE, Mini-Mental State Examination; NDR, no diabetic retinopathy; PPDR or PDR, pre-proliferative or proliferative diabetic retinopathy, or history of photocoagulation/vitreous surgery; SDR, simple diabetic retinopathy; TG, triglyceride; UA, uric acid

Variable	Value
Male	45 (58%)
Age	66 (59-70)
Disease duration (years)	14 (9-20)
BMI (kg/m²)	26.5 (22.8-30.4)
Smoking	
Never	29 (38%)
Former	36 (47%)
Current	12 (16%)
Type of diabetes	
Type 1	7 (9%)
Type 2	69 (90%)
Pancreatic	1 (1%)
Family history of diabetes	54 (70%)
Coronary artery disease	16 (21%)
Stroke	10 (13%)
Neuropathy	54 (70%)
Retinopathy (missing n = 6 (8%))	
NDR	41 (53%)
SDR	18 (23%)
PPDR or PDR	12 (16%)
Nephropathy	
1	36 (47%)
2	24 (31%)
3	15 (19%)
4	2 (3%)
5	0 (0%)
Diabetes treatment^†^	
No drug	6 (8%)
Oral medication	35 (45%)
GLP-1 analog	5 (6%)
Insulin	31 (40%)
Antihypertensive drug use	47 (61%)
Lipid-lowering drug use	50 (65%)
Systolic blood pressure (mmHg)	120 (112-132)
Diastolic blood pressure (mmHg)	66 (60-72)
HbA1c (%)	8.6 (7.7-10.1)
Fasting plasma glucose (mg/dL)	142 (117-184)
Alb (mg/dL)	4.1 (3.9-4.3)
UA (mg/dL)	5.4 (4.5-6.2)
TG (mg/dL)	119 (85-190)
LDL-C (mg/dL) (missing n = 5 (6%))	89 (72-119)^†^
BUN (mg/dL)	16.0 (13.6-20.0)
Cre (mg/dL)	0.84 (0.70-1.04)
eGFR (mL/min/1.73 m²)	67.6 (52.1-78.8)
CKD stage	
G1	6 (8%)
G2	43 (56%)
G3a	14 (18%)
G3b	12 (16%)
G4	2 (3%)
G5	0 (0%)
MMSE	29 (27-30)
HNA%	24.7 (22.0-27.6)

As indicated in Table 2, significant changes were observed in the following clinical parameters between baseline and the five-year follow-up point for all 77 participants: BMI, smoking status, coronary artery disease history, diabetic neuropathy, diabetic nephropathy, diabetes treatment status, lipid-lowering medication use, systolic blood pressure, diastolic blood pressure, HbA1c, creatinine, eGFR, CKD severity classification, and HNA%. Regarding diabetic complications, participants showed significant changes in diabetic neuropathy, eGFR, and CKD staging from baseline, likely attributable to aging and increased disease duration. As discussed in the following section, a detailed analysis of DR progression over the five-year follow-up period is presented, including a summary table showing the distribution of retinopathy stage transitions. We observed both improvement and deterioration in DR stages and conducted subgroup analyses accordingly.

**Table 2:** Clinical characteristics of 77 study participants at follow-up Values are expressed as medians (IQRs), and frequencies are presented as n (%). N = 77 (100%) unless otherwise specified. Statistical analyses were performed to determine the differences in each characteristic between baseline and five-year follow-up measurements. p-values derived from either a paired t-test or a Wilcoxon signed-rank test. The level of statistical significance was set at p < 0.05. * indicates p < 0.05; ** indicates p < 0.01; and *** indicates p < 0.001. ^†^ Participants using medications from multiple categories were classified into the lowest category within the classification hierarchy. Nephropathy stage was determined based on the Classification of Diabetic Nephropathy 2014 proposed by the Joint Committee on Diabetic Nephropathy in Japan. Alb, serum albumin; BUN, blood urea nitrogen; Cre, creatinine; eGFR, estimated glomerular filtration rate; GLP-1, glucagon-like peptide 1 receptor agonists; HbA1c, glycated hemoglobin; HNA%, percentage of human non-mercaptalbumin; LDL-C, low-density lipoprotein cholesterol; MMSE, Mini-Mental State Examination; NDR, no diabetic retinopathy; PPDR or PDR, pre-proliferative or proliferative diabetic retinopathy, or history of photocoagulation/vitreous surgery; SDR, simple diabetic retinopathy; TG, triglyceride; UA, uric acid

Variables	Value	Test statistics	p-value
Male	45 (58%)	Not applicable	Not applicable
Age	71 (64-76)	Not applicable	Not applicable
Disease duration (years)	19 (14-25)	Not applicable	Not applicable
BMI (kg/m²)	25.0 (22.4-28.9)	t = -3.51	<0.001^***^
Smoking		S = 187.5	0.024^*^
Never	27 (35%)		
Former	35 (45%)		
Current	15 (19%)		
Family history of diabetes	54 (70%)	Not applicable	Not applicable
Coronary artery disease	23 (30%)	S = 259	0.007^**^
Stroke	11 (14%)	S = 38.5	0.32
Neuropathy	65 (84%)	S = 396	<0.001^***^
Retinopathy (missing n = 9 (12%))		S = -22	0.85
NDR	41 (53%)		
SDR	14 (18%)		
PPDR or PDR	13 (17%)		
Nephropathy		S = 584.5	<0.001^***^
1	30 (39%)		
2	23 (30%)		
3	17 (22%)		
4	5 (6%)		
5	2 (3%)		
Diabetes treatment^†^		S = 420	0.016^*^
No drug	0 (0%)		
Oral medication	29 (38%)		
GLP-1 analog	16 (21%)		
Insulin	32 (42%)		
Antihypertensive drug use	54 (70%)	S = 245	0.07
Lipid-lowering drug use	59 (77%)	S = 319.5	0.012^*^
Systolic blood pressure (mmHg)	129 (120-140)	t = 3.61	<0.001^***^
Diastolic blood pressure (mmHg)	72 (64-80)	t = 3.85	<0.001^***^
HbA1c (%) (missing n = 2 (3%))	7.6 (7.1-8.3)	S = -899.5	<0.001^***^
Casual plasma glucose (mg/dL)	144 (119-193)	Not applicable	Not applicable
Alb (mg/dL)	4.2 (4.0-4.4)	t = 1.90	0.061
UA (mg/dL) (missing n = 1 (1%))	5.3 (4.3-6.3)	t = -0.33	0.74
TG (mg/dL)	128 (87-221)	t = 1.78	0.08
LDL-C (mg/dL)	85 (72-112)	t = -1.46	0.15
BUN (mg/dL) (missing n = 4 (5%))	17.3 (13.3-21.0)	t = 0.27	0.79
Cre (mg/dL)	0.89 (0.72-1.12)	t = 2.26	0.027^*^
eGFR (mL/min/1.73 m²)	60.1 (40.3-71.7)	t = -5.46	<0.001^***^
CKD stage		S = 733.5	<0.001^***^
G1	3 (4%)		
G2	36 (47%)		
G3a	18 (23%)		
G3b	13 (17%)		
G4	4 (5%)		
G5	3 (4%)		
MMSE	29 (28-30)	t = 1.07	0.29
HNA%	26.7 (24.3-31.9)	t = 5.72	<0.001^***^
Duration of follow-up	4.8 (4.6-5.0)	Not applicable	Not applicable

Based on these clinical findings, we proceeded to conduct an exploratory investigation of our hypothesis that baseline HNA% correlates with the progression of various diabetic complications. The subsequent analyses examined retinopathy, CKD staging, and macrovascular complications.

Exploratory analysis of baseline HNA% in relation to five-year changes in retinopathy

Among the 36 participants (47%) without retinopathy at baseline, nine (12%) showed deterioration at follow-up, while 27 (35%) remained unchanged. Of the 15 participants (19%) with SDR at baseline, 10 (13%) showed improvement at follow-up, while five (6%) showed no improvement (Table 3).

**Table 3:** Distribution of retinopathy classifications at baseline and five-year follow-up period Frequencies are presented as n (%). Cases without fundus examination data at either baseline or five-year follow-up were treated as missing. NDR, no diabetic retinopathy; PPDR/PDR, pre-proliferative or proliferative diabetic retinopathy, or history of photocoagulation/vitreous surgery; SDR, simple diabetic retinopathy

Baseline	Follow-up	Number of participants (n, %)
NDR	NDR	27 (35%)
NDR	SDR	8 (10%)
NDR	PPDR/PDR	1 (1%)
SDR	NDR	10 (13%)
SDR	SDR	5 (6%)
SDR	PPDR/PDR	0 (0%)
PPDR/PDR	PPDR/PDR	12 (16%)
Missing data		14 (18%)

**Figure 2:**
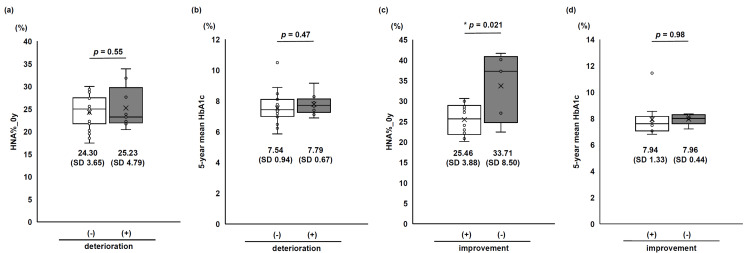
Baseline HNA% and five-year mean HbA1c in relation to retinopathy Comparisons of (a) baseline HNA% (HNA%_0y) and (b) five-year mean HbA1c between non-deteriorated and deteriorated groups among 36 participants (47%) with baseline NDR. Comparison of (c) baseline HNA% (HNA%_0y) and (d) five-year mean HbA1c between the improved group and the non-improved group among 15 participants with baseline SDR. Graphs show medians and IQRs, while data are expressed as mean (SD). p-values derived from Student’s t-test, with statistical significance set at p < 0.05. * indicates p < 0.05. “(+)” and “(-)” in the figure denote the presence or absence of deterioration/improvement. HbA1c, glycated hemoglobin; HNA, human non-mercaptalbumin; NDR, no diabetic retinopathy; SDR, simple diabetic retinopathy

Among the 36 participants (47%) without diabetic retinopathy (NDR) at baseline, we compared baseline HNA% (HNA%_0y) between the non-deteriorated group (deterioration (-)) and the deteriorated group (deterioration (+)). No statistically significant difference was observed (Figure 2a). Similarly, comparison of five-year mean HbA1c between these groups showed no statistically significant difference (Figure 2b). Among the 15 participants (19%) with SDR at baseline, we compared baseline HNA% (HNA%_0y) between the improved group (improvement (+)) and the non-improved group (improvement (-)). A statistically significant difference was observed, with the improved group showing lower values (improvement (+) group: 25.46 (SD 3.88) vs. improvement (-) group: 33.71 (SD 8.50), p = 0.021; Figure 2c). However, comparison of five-year mean HbA1c between these groups showed no statistically significant difference (Figure 2d). Furthermore, the non-improved group exhibited significantly longer disease duration compared with the improved group (Table 4).

**Table 4:** Characteristics of the participants with SDR at baseline, stratified by improvement +/- at follow-up Values are expressed as medians (IQRs), and frequencies are presented as n (%). N = 10 (13%) for the improvement (+) group and n = 5 (6%) for the improvement (-) group unless otherwise specified. Statistical analyses were performed to determine the differences in each characteristic between the improved and the non-improved groups. p-values derived from either Student’s t-test or Pearson’s chi-squared test. The level of statistical significance was set at p < 0.05. * indicates p < 0.05. ^†^ Participants using medications from multiple categories were classified into the lowest category within the classification hierarchy. ^‡ ^Missing data; n = 2 (3%). Nephropathy stage was determined based on the Classification of Diabetic Nephropathy 2014 proposed by the Joint Committee on Diabetic Nephropathy in Japan. Alb, serum albumin; BUN, blood urea nitrogen; Cre, creatinine; eGFR, estimated glomerular filtration rate; GLP-1, glucagon-like peptide 1 receptor agonists; HbA1c, glycated hemoglobin; HNA%_0y, percentage of human non-mercaptalbumin at baseline; LDL-C, low-density lipoprotein cholesterol; MMSE, Mini-Mental State Examination; NDR, no diabetic retinopathy; PPDR or PDR, pre-proliferative or proliferative diabetic retinopathy, or history of photocoagulation/vitreous surgery; SDR, simple diabetic retinopathy; TG, triglyceride; UA, uric acid

Variables	Improvement (+) (n = 10; 13%)	Improvement (-) (n = 5; 6%)	Test statistics	p-value
Male	9 (12%)	2 (3%)	χ² = 4.26	0.039^*^
Age	66 (58-70)	66 (65-76)	t = -1.13	0.28
Disease duration (years)	13 (10-16)	22 (16-36)	t = -2.98	0.011^*^
BMI (kg/m²)	26.1 (23.1-32.9)	26.5 (24.2-32.9)	t = -0.30	0.77
Smoking			χ² = 2.18	0.34
Never	0 (0%)	1 (1%)		
Former	7 (9%)	3 (4%)		
Current	3 (4%)	1 (1%)		
Family history of diabetes	7 (9%)	4 (5%)	χ² = 0.17	0.68
Coronary artery disease	4 (5%)	2 (3%)	χ² = 0.00	1
Stroke	1 (1%)	0 (0%)	χ² = 0.54	0.46
Neuropathy	6 (8%)	5 (6%)	χ² = 2.73	0.099
Nephropathy			χ² = 2.85	0.24
1	3 (4%)	2 (3%)		
2	4 (5%)	0 (0%)		
3	3 (4%)	3 (4%)		
4	0 (0%)	0 (0%)		
5	0 (0%)	0 (0%)		
Diabetes treatment^†^			χ² = 0.17	0.68
No drug	0 (0%)	0 (0%)		
Oral medication	3 (4%)	1 (1%)		
GLP-1 analog	0 (0%)	0 (0%)		
Insulin	7 (9%)	4 (5%)		
Antihypertensive drug use	7 (9%)	5 (6%)	χ² = 1.88	0.17
Lipid-lowering drug use	7 (9%)	3 (4%)	χ² = 0.15	0.7
Systolic blood pressure (mmHg)	132 (127-139)	134 (118-142)	t = 0.18	0.86
Diastolic blood pressure (mmHg)	67 (59-81)	60 (51-73)	t = 1.11	0.29
Baseline HbA1c (%)	8.8 (7.3-11.1)	8.6 (7.5-9.5)	t = 0.74	0.47
Five-year mean HbA1c (%)	7.6 (7.1-8.2)	8.0 (7.6-8.3)	t = -0.030	0.98
Fasting plasma glucose (mg/dL)	141 (113-196)	151 (110.5-186)	t = 0.12	0.91
Alb (mg/dL)	4.2 (3.9-4.4)	3.8 (3.8-4.0)	t = 1.97	0.07
UA (mg/dL)	5.1 (4.2-6.3)	6.6 (4.6-7.8)	t = -1.35	0.2
TG (mg/dL)	150 (104-412)	191 (95-248)	t = 0.77	0.45
LDL-C (mg/dL)	91 (62-104)‡	89 (84-129)	t = -1.09	0.3
BUN (mg/dL)	17.5 (14.8-20.0)	18.1 (15.0-30.0)	t = -1.44	0.17
eGFR (mL/min/1.73 m²)	59.0 (42.5-75.4)	53.7 (37.6-80.9)	t = 0.33	0.75
CKD stage			χ² = 0.60	0.9
G1	1 (1%)	0 (0%)		
G2	4 (5%)	2 (3%)		
G3a	2 (3%)	1 (1%)		
G3b	3 (4%)	2 (3%)		
G4	0 (0%)	0 (0%)		
G5	0 (0%)	0 (0%)		
MMSE	29 (27-30)	28 (27-30)	t = -0.12	0.91
HNA%_0y	25.7 (21.8-28.9)	37.3 (24.7-40.9)	t = -2.64	0.021^*^
Duration of follow-up	4.9 (4.7-5.0)	4.8 (4.3-5.0)	t = 0.98	0.34

These findings regarding the hypothesis that baseline HNA% predicts diabetic retinopathy prognosis yielded the following results: In the baseline NDR group, no statistically significant correlation was found between baseline HNA% levels and the development of retinopathy. However, in the baseline SDR group, baseline HNA% levels were associated with the duration of diabetes and predicted five-year retinopathy improvement, independent of HbA1c levels.

Exploratory analysis of baseline HNA% in relation to the five-year prognosis of renal function

The CKD severity classification stages at baseline and five-year follow-up for all 77 participants are presented in Table 1, Table 2, and Table 5. Of the 77 participants, 43 (56%) were classified as stage G2 at baseline. Among these, 12 (16%) showed deterioration at follow-up, while 31 (40%) remained stable.

**Table 5:** Distribution of CKD severity classifications stages at baseline and at the five-year follow-up period Frequencies are presented as n (%). CKD, chronic kidney disease.

Baseline	Follow-up	Number of participants (n, %)
1	1	1 (1%)
1	2	4 (5%)
1	3a	1 (1%)
2	1	2 (3%)
2	2	29 (38%)
2	3a	11 (14%)
2	3b	1 (1%)
3a	2	3 (4%)
3a	3a	6 (8%)
3a	3b	5 (6%)
3b	3b	7 (9%)
3b	4	4 (5%)
3b	5	1 (1%)
4	5	2 (3%)
Total		77 (100%)

**Figure 3:**
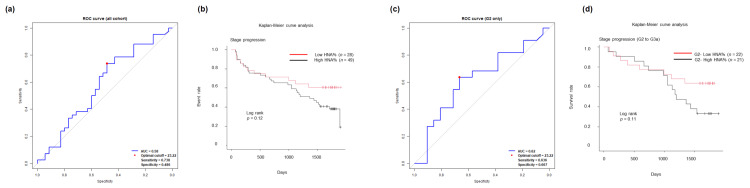
Baseline HNA% in relation to the five-year prognosis of renal function ROC analyses of baseline HNA% for CKD stage progression in (a) all 77 participants and (c) the 43 participants (56%) with CKD stage G2 at baseline. The optimal cutoff value was determined to be 23.22% in both analyses. The participants were stratified (b) into High HNA% (n = 49; 64%) and Low HNA% (n = 28; 36%) groups in the entire cohort and (d) into G2-High HNA% (n = 21; 27%) and G2-Low HNA% (n = 22; 29%) groups among 43 participants (56%) with CKD stage G2 at baseline, based on the cutoff threshold. Survival analyses for CKD stage progression were performed in (b) High vs. Low HNA% groups, and in (d) G2-High vs. G2-Low HNA% groups. p-values derived from the log-rank test, with statistical significance set at p < 0.05. AUC, area under the curve; CKD, chronic kidney disease; HNA, human non-mercaptalbumin; ROC, receiver operating characteristic

For all 77 participants, ROC analysis was performed to determine the optimal cutoff value of baseline HNA% for discriminating CKD severity stage progression. As shown in Figure 3a, the optimal cutoff value was determined to be 23.22% (sensitivity 73.8%, specificity 48.6%, AUC 0.58). The participants were stratified into High and Low HNA% groups based on this cutoff, and the incidence of renal function deterioration between baseline and follow-up was compared between the two groups. As shown in Figure 3b, the survival proportion (percentage without CKD severity stage progression) at 1,600 days, which was the shortest follow-up duration of all the participants, was 40.8% in the High group and 60.7% in the Low group, with a risk ratio of 1.51 favoring the Low group. While both groups showed similar progression patterns until day 500, the High group subsequently demonstrated more frequent stage progression. The overall analysis at the follow-up, but not the log-rank test, revealed a statistically significantly higher event occurrence rate in the High group (RR 1.61, p = 0.042, Table 6). However, there were significant differences in baseline CKD stages (Table 7), which could be a potential confounder.

**Table 6:** Contingency table of baseline HNA% levels (High/Low) and CKD stage progression events among all 77 participants Frequencies are presented as n (%). The participants were stratified into High and Low HNA% groups based on the cutoff value of 23.22%. A chi-squared test was performed to assess differences in the distribution between the two groups, and the corresponding p-value was calculated. Statistical significance was set at p < 0.05. CKD, chronic kidney disease; HNA%, percentage of human non-mercaptalbumin

Group	Event (-)	Event (+)	Total	Relative risk	Chi-squared value	p-value
High HNA%	18 (23%)	31 (40%)	49 (64%)	1.61	4.132	0.042
Low HNA%	17 (22%)	11 (14%)	28 (36%)			-
Total	35 (45%)	42 (55%)	77 (100%)			-

**Table 7:** Characteristics of all the participants at baseline, stratified by the baseline HNA% cutoff threshold Values are expressed as medians (IQRs), and frequencies are presented as n (%). The participants were stratified into High and Low HNA% groups based on the cutoff value of 23.22%. N = 49 (64%) for the High group or n = 28 (36%) for the Low group unless otherwise specified. Statistical analyses were performed to determine the differences in each characteristic between the two groups. p-values derived from either Student’s t-test or Pearson’s chi-squared test. The level of statistical significance was set at p < 0.05. * indicates p < 0.05; ** indicates p < 0.01; and *** indicates p < 0.001. ^†^ Participants using medications from multiple categories were classified into the lowest category within the classification hierarchy. Nephropathy stage was determined based on the Classification of Diabetic Nephropathy 2014 proposed by the Joint Committee on Diabetic Nephropathy in Japan. Alb, serum albumin; BUN, blood urea nitrogen; Cre, creatinine; eGFR, estimated glomerular filtration rate; GLP-1, glucagon-like peptide 1 receptor agonists; HbA1c, glycated hemoglobin; HNA%_0y, percentage of human non-mercaptalbumin at baseline; LDL-C, low-density lipoprotein cholesterol; MMSE, Mini-Mental State Examination; NDR, no diabetic retinopathy; PPDR or PDR, pre-proliferative or proliferative diabetic retinopathy, or history of photocoagulation/vitreous surgery; RASi, renin-angiotensin system inhibitors; SDR, simple diabetic retinopathy; SGLT2i, sodium-glucose cotransporter-2 inhibitors; TG, triglyceride; UA, uric acid

Variables	Participants with High HNA%_0y (n = 49; 64%)	Participants with Low HNA%_0y (n = 28; 36%)	Test statistics	p-value
Male	28 (36%)	17 (22%)	χ² = 0.094	0.76
Age	66 (59-74)	64 (58-69)	t = -1.15	0.25
Disease duration (years)	14 (9-22)	14 (8-18)	t = -0.79	0.43
BMI (kg/m²)	26.9 (23.6-32.6)	25.2 (21.6-30.0)	t = -1.20	0.23
Smoking			χ² = 4.83	0.09
Never	17 (22%)	12 (16%)		
Former	27 (35%)	9 (12%)		
Current	5 (6%)	7 (9%)		
Family history of diabetes	33 (43%)	21 (27%)	χ² = 0.50	0.48
Coronary artery disease	10 (13%)	6 (8%)	χ² = 0.011	0.92
Stroke	8 (10%)	2 (3%)	χ² = 1.33	0.25
Neuropathy	39 (51%)	15 (19%)	χ² = 5.76	0.016^*^
Retinopathy (missing n = 3 (4%), High; n = 3 (4%), Low)			χ² = 2.18	0.34
NDR	25 (32%)	16 (21%)		
SDR	11 (14%)	7 (9%)		
PPDR or PDR	10 (13%)	2 (3%)		
Nephropathy			χ² = 2.86	0.41
1	20 (26%)	16 (21%)		
2	16 (21%)	8 (10%)		
3	11 (14%)	4 (5%)		
4	2 (3%)	0 (0%)		
5	0 (0%)	0 (0%)		
Diabetes treatment^†^			χ² = 6.51	0.089
No drug	5 (6%)	1 (1%)		
Oral medication	17 (22%)	18 (23%)		
GLP-1 analog	4 (5%)	1 (1%)		
Insulin	23 (30%)	8 (10%)		
Antihypertensive drug use	32 (42%)	15 (19%)	χ² = 1.03	0.31
Lipid-lowering drug use	30 (39%)	20 (26%)	χ² = 0.82	0.37
Systolic blood pressure (mmHg)	120 (111-133)	120 (111-130)	t = -0.030	0.98
Diastolic blood pressure (mmHg)	64 (59-72)	68 (63-74)	t = 1.30	0.2
Baseline HbA1c (%)	8.6 (7.6-9.9)	8.7 (7.8-10.6)	t = 0.45	0.66
Five-year mean HbA1c (%) (missing n = 1 (1%), Low)	7.6 (7.4-8.2)	7.7 (7.1-8.2)^†^	t = -0.78	0.44
Fasting plasma glucose (mg/dL)	140 (113-179)	145 (123-201)	t = 0.73	0.47
Alb (mg/dL)	4.1 (3.9-4.3)	4.2 (4.0-4.4)	t = 1.14	0.26
UA (mg/dL)	5.6 (4.6-6.6)	4.9 (4.4-6.1)	t = -1.32	0.19
TG (mg/dL)	115 (84-167)	153 (85-247)	t = 1.47	0.15
LDL-C (mg/dL) (missing n = 3 (4%), High; n = 2 (3%), Low)	89 (68-118)	90 (77-122)	t = 0.26	0.79
BUN (mg/dL)	18.1 (14.0-22.0)	15.0 (12.1-18.4)	t = -1.89	0.063
eGFR (mL/min/1.73 m²)	59.7 (41.7-74.2)	76.4 (66.6-84.1)	t = 3.76	<0.001^***^
CKD stage			χ² = 13.9	0.008^**^
G1	3 (4%)	3 (4%)		
G2	21 (27%)	22 (29%)		
G3a	11 (14%)	3 (4%)		
G3b	12 (16%)	0 (0%)		
G4	2 (3%)	0 (0%)		
G5	0 (0%)	0 (0%)		
MMSE	29 (28-30)	29 (27-30)	t = 0.15	0.88
HNA%_0y	27.2 (24.9-29.1)	20.8 (19.5-22.2)	t = -8.60	<0.001^***^
Duration of follow-up	4.8 (4.6-5.0)	4.8 (4.5-5.0)	t = 0.39	0.7
Addition of SGLT2i	12 (16%)	10 (13%)	χ² = 1.57	0.46
Addition of RASi	3 (4%)	7 (9%)	χ² = 7.91	0.019^*^

Therefore, to ensure comparable backgrounds between the two groups, we conducted a similar analysis focusing on 43 participants (56%) with CKD stage G2 at baseline, a cohort that comprises more than half of the participants. As shown in Figure 3c, ROC analysis determined the optimal cutoff value to be 23.22% (sensitivity 63.6%, specificity 66.7%, AUC 0.62), coinciding with that for the entire cohort. The participants were stratified into G2-High and G2-Low groups based on the cutoff. As shown in Figure 3d, the survival proportion at 1,600 days was 33.3% in the G2-High group and 63.6% in the G2-Low group, with a risk ratio of 1.83. While both groups showed similar progression patterns until day 1,000, the G2-High group demonstrated more frequent progression after the 1,000-day point. The overall analysis at follow-up, but not the log-rank test, revealed a statistically significantly higher event occurrence rate in the G2-High group (RR 1.83, p = 0.047, Table 8). Comparison of baseline nephropathy categories between the groups showed no significant difference (Table 9).

**Table 8:** Contingency table of baseline HNA% levels (G2-High vs. G2-Low) and CKD stage progression among 43 participants (56%) with baseline CKD stage G2 Frequencies are presented as n (%). The participants were stratified into G2-High and G2-Low HNA% groups based on the cutoff value of 23.22%. A chi-squared test was performed to assess differences in the distribution between the two groups, and the corresponding p-value was calculated. Statistical significance was set at p < 0.05. CKD, chronic kidney disease; HNA%, percentage of human non-mercaptalbumin

Group	Event (-)	Event (+)	Total	Relative risk	Chi-squared value	p-value
G2-High	7 (9%)	14 (18%)	21 (27%)	1.83	3.949	0.047
G2-Low	14 (18%)	8 (10%)	22 (29%)			-
Total	21 (27%)	22 (29%)	43 (56%)			-

**Table 9:** Characteristics of 43 participants (56%) with CKD stage G2 at baseline, stratified by the HNA%_0y cutoff threshold Values are expressed as medians (IQRs), and frequencies are presented as n (%). N = 21 (27%) for the G2-High group or n = 22 (29%) for the G2-Low group unless otherwise specified. The participants were stratified into G2-High and G2-Low HNA% groups based on the cutoff value of 23.22%. Statistical analyses were performed to determine the differences in each characteristic between the two groups. p-values derived from either Student’s t-test or Pearson’s chi-squared test. The level of statistical significance was set at p < 0.05. * indicates p < 0.05; ** indicates p < 0.01; and *** indicates p < 0.001. ^†^ Participants using medications from multiple categories were classified into the lowest category within the classification hierarchy. Nephropathy stage was determined based on the Classification of Diabetic Nephropathy 2014 proposed by the Joint Committee on Diabetic Nephropathy in Japan. Alb, serum albumin; BUN, blood urea nitrogen; CKD, chronic kidney disease; Cre, creatinine; eGFR, estimated glomerular filtration rate; GLP-1, glucagon-like peptide 1 receptor agonists; HbA1c, glycated hemoglobin; HNA%_0y, percentage of human non-mercaptalbumin at baseline; LDL-C, low-density lipoprotein cholesterol; MMSE, Mini-Mental State Examination; NDR, no diabetic retinopathy; PPDR or PDR, pre-proliferative or proliferative diabetic retinopathy, or history of photocoagulation/vitreous surgery; RASi, renin-angiotensin system inhibitors; SDR, simple diabetic retinopathy; SGLT2i, sodium-glucose cotransporter-2 inhibitors; TG, triglyceride; UA, uric acid

Variables	Participants with high HNA%_0y (G2-High, n = 21 (27%))	Participants with lower HNA%_0y (G2-Low, n = 22 (29%))	test statistics	p-value
Male	15 (19%)	12 (16%)	χ² = 1.31	0.25
Age	64 (56-72)	63 (58-68)	t = -0.070	0.94
Disease duration (years)	13 (8-20)	14 (8-19)	t = 0.013	0.99
BMI (kg/m²)	26.9 (22.7-28.8)	24.8 (21.4-28.3)	t = -0.48	0.63
Smoking			χ² = 2.84	0.24
Never	7 (9%)	11 (14%)		
Former	11 (14%)	6 (8%)		
Current	3 (4%)	5 (6%)		
Family history of diabetes	11 (14%)	18 (23%)	χ² = 4.24	0.040^*^
Coronary artery disease	2 (3%)	4 (5%)	χ² = 0.67	0.41
Stroke	3 (4%)	2 (3%)	χ² = 0.28	0.6
Neuropathy	15 (19%)	11 (14%)	χ² = 2.06	0.15
Retinopathy (missing n = 3 (4%), G2-High; n = 3 (4%), G2-Low)			χ² = 3.59	0.17
NDR	11 (14%)	15 (19%)		
SDR	4 (5%)	4 (5%)		
PPDR or PDR	3 (4%)	0 (0%)		
Nephropathy			χ² = 1.13	0.57
1	11 (14%)	15 (19%)		
2	7 (9%)	5 (6%)		
3	3 (4%)	2 (3%)		
4	0 (0%)	0 (0%)		
5	0 (0%)	0 (0%)		
Diabetes treatment^†^			χ² = 4.71	0.19
No drug	4 (5%)	1 (1%)		
Oral medication	7 (9%)	14 (18%)		
GLP-1 analog	1 (1%)	1 (1%)		
Insulin	9 (12%)	6 (8%)		
Antihypertensive drug use	10 (13%)	11 (14%)	χ² = 0.024	0.88
Lipid-lowering drug use	10 (13%)	15 (19%)	χ² = 1.87	0.17
Systolic blood pressure (mmHg)	116 (109-132)	120 (110-129)	t = 0.22	0.83
Diastolic blood pressure (mmHg)	60 (59-69)	69 (62-76)	t = 1.77	0.085
Baseline HbA1c (%)	8.8 (7.6-9.4)	8.7 (7.7-10.6)	t = 0.35	0.73
Five-year mean HbA1c (%) (missing n = 1 (1%), G2-Low)	7.6 (7.2-8.1)	7.6 (7.0-8.2)	t = -0.55	0.59
Fasting plasma glucose (mg/dL)	147 (116-191)	146 (119-205)	t = 0.68	0.50
Alb (mg/dL)	4.1 (4.0-4.4)	4.2 (4.0-4.4)	t = 0.35	0.73
UA (mg/dL)	5.5 (3.8-6.9)	4.7 (4.3-6.0)	t = -1.06	0.29
TG (mg/dL)	91 (70-129)	142 (72-247)	t = 2.34	0.024^*^
LDL-C (mg/dL) (missing n = 2 (3%), G2-Low)	105 (81-125)	90 (74-120)	t = -0.43	0.67
BUN (mg/dL)	15.7 (13.5-19.4)	14.3 (12.0-16.2)	t = -0.81	0.42
eGFR (mL/min/1.73 m²)	73.9 (67.0-78.8)	76.4 (68.2-83.8)	t = 1.06	0.29
MMSE	28 (27-30)	29 (27-30)	t = 0.39	0.70
HNA%	27.0 (24.6-27.8)	20.8 (19.4-22.2)	t = -8.06	<0.001^***^
Duration of follow-up	4.7 (4.6-4.9)	4.8 (4.5-5.0)	t = 0.36	0.72
Addition of SGLT2i	6 (8%)	6 (8%)	χ² = 0.009	0.92
Addition of RASi	2 (3%)	4 (5%)	χ² = 3.26	0.20

These results indicated that both in the overall cohort and in the baseline stage G2 subgroup, groups with baseline HNA% above the cutoff showed statistically significantly higher rates of renal function deterioration events. This suggested that baseline HNA% could predict renal function deterioration over a five-year period.

Exploratory analysis of HNA% in relation to the five-year onset of macrovascular complications

**Figure 4:**
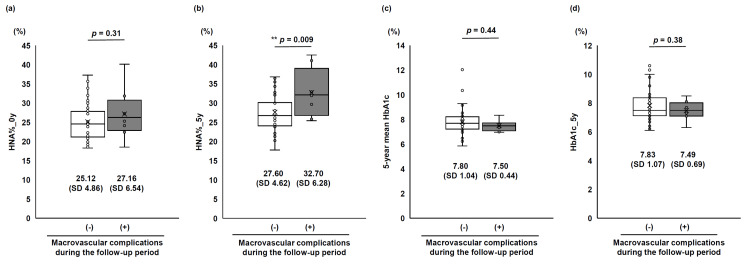
HNA% and HbA1c in relation to five-year onset of macrovascular complications Comparisons of (a) baseline HNA% (HNA%_0y), (b) follow-up HNA% (HNA%_5y), (c) five-year mean HbA1c, and (d) follow-up HbA1c (HbA1c_5y) between participants who did and did not develop macrovascular complications during the five-year follow-up period, among those free of macrovascular complications at baseline. Graphs show medians and IQRs, while data are expressed as mean (SD). p-values derived from Student’s t-test, with statistical significance set at p < 0.05. ** indicates p < 0.01. HbA1c, glycated hemoglobin; HNA, human non-mercaptalbumin

Among the study participants, the incidence of macrovascular complications during the five-year follow-up period was relatively low, with seven cases (9%) of coronary artery disease and one case (1%) of cerebrovascular disease (Table 1, Table 2). Therefore, these complications were analyzed collectively as macrovascular complications to evaluate whether HNA% could predict their onset. To examine the relationship between baseline HNA% and the development of macrovascular complications over the five-year period, we classified participants without baseline macrovascular complications into groups with and without incident complications (Table 10). We compared their baseline HNA% (HNA%_0y) between the non-incident and incident groups, and no statistically significant difference was observed (Figure 4a).

**Table 10:** Characteristics of 53 participants (69%) without macrovascular disease at baseline, stratified by the presence or absence of incident cases during the follow-up period Values are expressed as medians (IQRs), and frequencies are presented as n (%). N = 8 (10%) for the incident group and n = 45 (58%) for the non-incident group unless otherwise specified. Statistical analyses were performed to determine the differences in each characteristic between the two groups. p-values derived from either Student’s t-test or Pearson’s chi-squared test. The level of statistical significance was set at p < 0.05. * indicates p < 0.05. † Participants using medications from multiple categories were classified into the lowest category within the classification hierarchy. Nephropathy stage was determined based on the Classification of Diabetic Nephropathy 2014 proposed by the Joint Committee on Diabetic Nephropathy in Japan. Alb, serum albumin; BUN, blood urea nitrogen; CKD, chronic kidney disease; Cre, creatinine; eGFR, estimated glomerular filtration rate; GLP-1, glucagon-like peptide 1 receptor agonists; HbA1c, glycated hemoglobin; HNA%, percentage of human non-mercaptalbumin; LDL-C, low-density lipoprotein cholesterol; MMSE, Mini-Mental State Examination; NDR, no diabetic retinopathy; PPDR or PDR, pre-proliferative or proliferative diabetic retinopathy, or history of photocoagulation/vitreous surgery; RASi, renin-angiotensin system inhibitors; SDR, simple diabetic retinopathy; SGLT2i, sodium-glucose cotransporter-2 inhibitors; TG, triglyceride; UA, uric acid

Variables	Participants with incident cases during the follow-up period (n = 8; 10%)	Participants without incident cases during the follow-up period (n = 45; 58%)	Test statistics	p-value
Male	4 (5%)	26 (34%)	χ² = 0.17	0.68
Age	70.5 (63-73.5)	63 (56-69)	t = 2.17	0.035^*^
Disease duration (years)	9 (1.5-17)	12 (7-17)	t = -0.72	0.48
BMI (kg/m²)	26.9 (24.5-28.3)	25.4 (21.8-30.8)	t = -0.007	0.99
Smoking			χ² = 0.89	0.64
Never	2 (3%)	17 (22%)		
Former	5 (6%)	20 (26%)		
Current	1 (1%)	8 (10%)		
Family history of diabetes	5 (6%)	32 (42%)	χ² = 0.24	0.62
Neuropathy	5 (6%)	32 (42%)	χ² = 0.24	0.62
Retinopathy (missing n = 5 (6%), non-incident)			χ² = 2.28	0.32
NDR	6 (8%)	22 (29%)		
SDR	2 (3%)	9 (12%)		
PPDR or PDR	0 (0%)	9 (12%)		
Nephropathy			χ² = 6.18	0.1
1	3 (4%)	24 (31%)		
2	1 (1%)	14 (18%)		
3	4 (5%)	6 (8%)		
4	0 (0%)	1 (1%)		
5	0 (0%)	0 (0%)		
Diabetes treatment^†^			χ² = 1.28	0.73
No drug	1 (1%)	4 (5%)		
Oral medication	5 (6%)	21 (27%)		
GLP-1 analog	0 (0%)	3 (4%)		
Insulin	2 (3%)	17 (22%)		
Antihypertensive drug use	7 (9%)	23 (30%)	χ² = 3.66	0.056
Lipid-lowering drug use	5 (6%)	27 (35%)	χ² = 0.018	0.89
Systolic blood pressure (mmHg)	123 (114-140)	120 (110-130)	t = 0.81	0.42
Diastolic blood pressure (mmHg)	64 (60.5-76)	66 (60-74)	t = -0.10	0.92
Baseline HbA1c (%)	8.6 (7.3-9.7)	8.7 (7.7-9.8)	t = -0.038	0.97
Five-year mean HbA1c (%) (missing n = 1 (1%), non-incident)	7.5 (7.1-7.7)	7.7 (7.2-8.2)^†^	t = -0.79	0.44
Fasting plasma glucose (mg/dL)	116 (109-167)	145 (121-198)	t = -1.00	0.32
Alb (mg/dL)	4.3 (4.1-4.5)	4.1 (3.9-4.3)	t = 1.21	0.23
UA (mg/dL)	6.6 (5.4-7.1)	5.6 (4.4-6.2)	t = 1.73	0.09
TG (mg/dL)	193 (159-228)	125 (79-192)	t = 1.40	0.17
LDL-C (mg/dL)	105 (73-116)	92 (81-120)	t = -0.11	0.91
BUN (mg/dL)	19.3 (15.9-22.1)	15.9 (12.8-19.1)	t = 1.23	0.22
eGFR (mL/min/1.73 m²)	63.7 (47.2-69.1)	72.3 (54.1-79.9)	t = -0.98	0.33
CKD stage			χ² = 5.38	0.25
G1	0 (0%)	4 (5%)		
G2	4 (5%)	28 (36%)		
G3a	3 (4%)	4 (5%)		
G3b	1 (1%)	8 (10%)		
G4	0 (0%)	1 (1%)		
G5	0 (0%)	0 (0%)		
MMSE	28 (27-30)	29 (28-30)	t = -0.85	0.4
HNA%	26.3 (22.9-30.8)	24.5 (21.2-27.9)	t = 1.04	0.31
Duration of follow-up	4.8 (4.6-5.0)	4.8 (4.6-4.9)	t = 0.22	0.82
Addition of SGLT2i	2 (3%)	15 (19%)	χ² = 0.22	0.64
Addition of RASi	2 (3%)	6 (8%)	χ² = 1.17	0.56

Although the primary hypothesis regarding macrovascular complications of this study was not proven, we conducted additional exploratory analyses to gain further insights between the two groups, examining follow-up HNA% (HNA%_5y), mean HbA1c, and follow-up HbA1c (HbA1c_5y). The comparison of HNA% at the five-year follow-up point (HNA%_5y) showed significantly higher values in the incident group (non-incident group: 27.60% (SD 4.62), incident group: 32.70% (SD 6.28), p = 0.009; Figure 4b). No statistically significant differences were observed in either HbA1c at the five-year follow-up point (HbA1c_5y, Figure 4c) or five-year mean HbA1c (Figure 4d).

These findings suggested that while baseline HNA% could not predict the development of macrovascular complications, recent HNA% levels might be associated with their occurrence, irrespective of HbA1c levels.

## Discussion

Our findings suggest that baseline HNA% may be partially associated with the prognosis of diabetic microvascular complications over a five-year period. Baseline HNA% significantly correlated with the duration of diabetes in participants with SDR and with improvement of retinopathy in follow-up duration. Notably, there were no significant differences in HbA1c levels over the follow-up duration between the improved and non-improved groups. This finding carries important clinical implications, suggesting that baseline HNA% could be a historical indicator of glucose management, reflecting cumulative oxidative stress exposure over time. This may explain its predictive ability for retinopathy improvement independent of HbA1c levels. On the other hand, in the subgroup of NDR at baseline, HNA% did not significantly correlate with the development of diabetic retinopathy. This may be attributed to insufficient statistical power, as the number of participants who developed retinopathy was limited in this pilot study.

Baseline HNA% implied predictive value for renal function deterioration over the subsequent five-year period. For renal function analysis, we stratified all 77 cases into two groups based on the baseline HNA% cutoff value. While there were no significant differences in the frequency distribution of baseline diabetic nephropathy categories between groups, we observed significant differences in the overall incidence of renal function deterioration events. The survival time analysis did not show statistical significance, possibly due to minimal differences between groups up to approximately 1,000 days and the absence of proportional hazards. A subgroup analysis of 43 participants (56%) with CKD stage G2 at baseline, conducted to control for initial CKD severity, yielded similar results.

Evidence for quantitative oxidative markers in the assessment of diabetic complications remains limited [13,18-20]. Previous studies have shown that oxidized albumin levels increase in liver failure [21,22] and renal failure [23,24], suggesting its potential as a marker of systemic oxidative status. In people with diabetes, our group’s previous work demonstrated that HNA% correlates cross-sectionally with the presence and stages of neuropathy, retinopathy, and nephropathy [16]. Fukuhara et al. reported that among 126 elderly patients with diabetes, HNA% correlated with the progression of retinopathy and nephropathy, showed higher values in hypertensive groups, and served as a strong predictor of ADL status [25]. Longitudinal studies include Soejima et al.’s 2004 report on 13 dialysis patients, showing HNA fraction reduction from 38.2% (SD 8.2%) to 21.7% (SD 8.0%) after dialysis [23]. In 2010, Terawaki et al. followed 86 dialysis patients for two years, finding that HNA values were associated with cardiovascular disease (CVD) onset and mortality [26]. In 2021, Imafuku et al. conducted a retrospective case-control study with a two-year follow-up, demonstrating that patients with Cys-albumin (equivalent to oxidized albumin) above the cutoff value (25.69%) showed significantly faster progression from CKD stage G2 to G3a compared to those below the cutoff [17].

This pilot study offers novel insight by conducting an exploratory longitudinal analysis of the association between HNA%, a serum marker of oxidative stress, and the prognosis of diabetic retinopathy. Given that HNA% can be measured rapidly and precisely, its clinical utility may lie in supporting physicians to recommend ophthalmologic evaluations, especially considering the current report that many patients with diabetes do not undergo regular ophthalmic checkups [27]. Moreover, this study also implied a correlation between baseline HNA% and the progression of CKD over a five-year follow-up period, longer than the previous report [17]. Our definition of CKD progression as stage deterioration confirmed by two consecutive regular blood tests from baseline allowed for more accurate assessment of chronic disease progression, distinguishing it from temporary renal function deterioration due to dehydration or acute illness.

A notable difference from the previous report is the absence of significant differences between groups at the two-year follow-up period. This may be attributed to three factors: First, the previous study measured Cys-albumin, which differs methodologically from our oxidized albumin measurements. Second, our cutoff value of 23.22% was slightly lower than the previously reported 25.69%. Third, our overall event rate of about 40% was lower than the over 50% reported previously, possibly due to our more stringent definition of CKD progression requiring two consecutive confirmatory tests. While both previous and current studies provide valuable insights, they differ in their temporal predictions of renal function deterioration. Prospective studies are needed to further validate these findings.

Regarding macrovascular complications, including cerebrovascular and coronary artery diseases, HNA% at the follow-up period, but not baseline levels, significantly correlated with the incidence of these complications during the follow-up, despite comparable HbA1c levels. Terawaki et al. previously reported on the relationship between oxidized/reduced albumin and macrovascular complications in 2010 [26]. In their two-year follow-up of 86 dialysis patients, 20 patients experienced macrovascular events. The CVD onset group showed lower reduced albumin fractions both pre- and post-dialysis, consistent with higher HNA%. Moreover, patients with a pre-dialysis HNA fraction ≥60% showed an adjusted odds ratio for CVD onset of 5.0 (95% CI: 1.2-21.3), while those with post-dialysis HNA fraction ≥ 40% showed an adjusted odds ratio of 20.6 (95% CI: 3.2-134.7). However, the predictive value of HNA% for macrovascular complications in people with diabetes has not been previously reported.

A few hypotheses might explain the finding on macrovascular complications in this pilot study: regular HNA% monitoring might reveal that sudden increases in HNA% correlate with the new onset of macrovascular complications, or the onset of macrovascular complications might have resulted in elevated HNA%. To test these hypotheses, prospective observational studies incorporating regular HNA% measurements are warranted.

Limitations

This study has several limitations. First, selection bias may exist as all participants were included from a single academic hospital and had a previous hospitalization history, potentially overrepresenting participants with longer disease duration. Second, survival bias may be present as only 77 participants could be included and analyzed retrospectively during the follow-up period, which coincided with the global COVID-19 pandemic. Third, being a retrospective study, information about supplement and antioxidant intake was unavailable. Fourth, while most cases were type 2 diabetes, we could not adequately examine type 1 diabetes due to insufficient sample sizes. Lastly, due to the limited sample size, the statistical power of this study may have been insufficient to detect subtle associations. Findings should be interpreted as exploratory, and multivariate analysis could not be performed.

## Conclusions

This pilot study provides preliminary evidence that lower baseline HNA% was associated with shorter duration of diabetes and with five-year improvement of retinopathy in participants with SDR at baseline, independent of HbA1c levels. On the other hand, in the subgroup of NDR at baseline, baseline HNA% was not significantly associated with the onset of diabetic retinopathy. We also observed significant differences in the overall incidence of renal function deterioration events between the high HNA% group and the low HNA% group at baseline. HNA% at the follow-up period, but not at baseline levels, significantly correlated with the incidence of macrovascular complications during the follow-up, despite comparable HbA1c levels. To establish more generalizable conclusions, prospective studies are required.
